# Hybrid operation technique for incisional hernia repair: a systematic review and meta-analysis of intra- and postoperative complications

**DOI:** 10.1007/s10029-021-02497-3

**Published:** 2021-09-18

**Authors:** L. Matthijs Van den Dop, Gijs H. J. De Smet, Gert-Jan Kleinrensink, Willem E. Hueting, Johan F. Lange

**Affiliations:** 1 Department of Surgery, Erasmus University Medical Center, Room Ee-173, Post box 2040, 3000 Rotterdam, CA The Netherlands; 2 Department of Neuroscience, Erasmus University Medical Center, Rotterdam, The Netherlands; 3 Department of Surgery, Alrijne Ziekenhuis, Leiderdorp, Leiden, The Netherlands; 4 Department of Surgery, IJsselland Ziekenhuis, Capelle Aan Den IJssel, The Netherlands

**Keywords:** Hybrid, Incisional hernia, Laparoendoscopic, Surgical technique, Postoperative complications

## Abstract

**Background:**

Incisional hernia (IH) occurs approximately in 15% of patients after midline surgery. Surgical treatment for IHs include a solely open or solely laparoscopic approach with mesh placement. Recently, hybrid (combined laparoscopic and open) approaches have been introduced. This systematic review evaluates perioperative complications of hybrid incisional hernia repair (HIHR).

**Methods:**

EMBASE, Medline via OvidSP, Web of Science, Cochrane and Google Scholar databases were searched. Studies providing data on intra- and postoperative complications in patients who underwent HIHR were included. Data on intra- and postoperative complications were extracted and meta-analyses were performed. Study quality was assessed with the Newcastle Ottowa Scale, ROBINS-I tool, and Cochrane risk of bias. PROSPERO registration: CRD42020175053.

**Results:**

Eleven studies (*n* = 1681 patients) were included. Five studies compared intra-operative complications between HIHR and laparoscopic incisional hernia repair (LIHR) with a pooled incidence of 1.8% in HIHR group and 2.8% in LIHR group (*p* = 0.13). Comparison of postoperative prevalence of surgical site occurrences (SSOs) (23% versus 26%, *p* = 0.02) and surgical site occurrences requiring interventions (SSOPIs) (1.5% versus 4.1%, *p* < 0.01) were in favour of the HIHR group. Overall postoperative complications seemed to occur less frequent in the HIHR group, though no hard statements could be made due to the vast heterogeneity in reporting between studies.

**Conclusion:**

Although the majority of studies were retrospective and included a small number of patients, HIHR seemingly led to less SSOs and SSOPIs. This systematic review forms a strong invitation for more randomized controlled trials to confirm the benefits of this approach.

**Supplementary Information:**

The online version contains supplementary material available at 10.1007/s10029-021-02497-3.

## Introduction

Incisional hernia (IH) can occur after any type of abdominal incision. The midline incision is most prone for IH development [1]. In the normal population, approximately 15% of patients develop IH after midline surgery, though in high-risk patients, incidences as high as 40% have been reported [2–4]. Patients suffering from IH can experience pain, impaired function in activities in daily life, and aesthetic dissatisfaction due to the bulging at the site of the hernia [5]. For some patients, watchful waiting is a justifiable treatment option due to risks associated with surgical complications. However, quality of life impairment and risk for incarceration and strangulation of abdominal contents (i.e. greater omentum, fat or bowel) may warrant surgical restoration of the abdominal wall [5].

To achieve anatomical restoration of the abdominal wall, two approaches are considered conventional: open incisional hernia repair (OIHR) and laparoscopic incisional hernia repair (LIHR). Comparing OIHR and the minimally invasive LIHR, a number of advantages and disadvantages can be distinguished. Advantages of the LIHR are reported to be as follows: shorter length of stay (LOS), less postoperative pain, and fewer postoperative complications [6–9]. Advantageous aspects of OIHR include complete hernia sac resection and multiple mesh positioning options. Disadvantages of LIHR include higher rates of enterotomies or other intra-operative complications, higher costs, higher rate of seroma formation, bulging of the mesh, and longer operation time [10, 11]. Recently, a novel approach is being used in the field of IH repair: the hybrid procedure. A hybrid procedure combines the laparoscopic and open approaches and hereby endeavors to minimize disadvantages of both procedures, while maintaining the advantages.

To date, there is no systematic review or meta-analysis on the subject of hybrid incisional hernia repair (HIHR), and whether these theoretical advantages are applicable to daily surgical practice. The aim of this study was to compare intra- and postoperative complications of HIHR with LIHR and provide an outlook on neoteric prospects in IH repair.

## Methods

The study protocol was registered in PROSPERO (CRD42020175053; International Prospective Register of Systematic Reviews). The Preferred Reporting Items for Systematic Reviews and Meta-Analyses (PRISMA) statement [12] and the Meta-Analysis of Observational Studies in Epidemiology (MOOSE) guideline [13] were followed. The article by Wille-Jørgensen et al*.* on systematic reviews and meta-analyses in coloproctology was used for methodological guidance [14].

### In- and exclusion criteria

Randomized and non-randomized clinical trials were included. Furthermore, prospective, or retrospective cohort or case–control studies providing data on intra- and postoperative complications in patients that underwent hybrid procedure for IH repair were included. IH was defined by the EuraHS working group as “a ventral hernia that developed after surgical trauma to the abdominal wall, including recurrences after repair of primary ventral hernias” [15]. A hybrid procedure for IH repair was characterized by combining a laparoscopic approach and a (mini-)laparotomy within one procedure. Case reports, reviews, letters, presentations, abstracts or comments were excluded. Studies were also excluded when patients were < 18 years of age, studies included less than ten patients, no clear description of the HIHR technique was reported, no information of any intra- or postoperative complications was reported, or the article was not written in the English language.

### Search strategy

A systematic search was performed by a biomedical information specialist of the Erasmus University Medical Center Library with input and assistance of the first author (L.M.v.d.D.). EMBASE, Medline via OvidSP, Web of Science, Cochrane and Google Scholar databases were searched on the 1st of February 2021. There was no limit in publication date. Full search syntax and number of articles per database are shown in the Supplemental. After removal of duplicates, the identified articles were reviewed independently by two reviewers (L.M.v.d.D. and G.H.J.d.S.) on title and abstract, followed by full-text review using EndNote X9^®^. Any discrepancies in article selection were discussed, and decision for in- or exclusion was made when consensus was reached between both reviewers. All relevant references in the included studies were checked manually to investigate whether the search syntax was correct and to ensure additional studies were not missed.

### Data extraction

Data extraction of all included studies was performed independently by two reviewers (L.M.v.d.D. and G.H.J.d.S.) using standard forms covering study characteristics (year, study design, number of patients), patient characteristics (sex, age, body mass index (BMI)), hernia characteristics (type, location, size), surgical characteristics (description of procedure, mesh position, mesh type, mesh fixation, length of stay, operation time), intra-operative complications (enterotomy, bleeding, bladder injury), and postoperative complications (seroma, surgical site occurrences (SSO), SSOs requiring procedural interventions (SSOPI), undetectable bowel injury, readmission, reoperation, mortality). Differences in data selection of each study were discussed among both reviewers until a decision could be made about the correct data. In case of uncertainties on reported study data, the corresponding author was contacted when possible.

### Study quality assessment

Two reviewers (L.M.v.d.D. and G.H.J.d.S.) independently assessed the quality of included studies by assessing the level of evidence according to the Oxford Centre for Evidence-based Medicine Levels of Evidence [16]. The Risk Of Bias In Non-randomised Studies–of Interventions (ROBINS-I) tool [17] was assessed in non-randomized studies, and the Cochrane risk of bias tool [18] in randomized studies.

### Statistical analysis

All analyses were performed with Review Manager 5.3 (Nordic Cochrane Centre, Copenhagen, Denmark). Pooled odds ratios (ORs) were calculated using the Mantel–Haenszel random effects model. The effect of each included study on the meta-analysis was examined by removing studies one at a time in order to calculate whether one study would change the significance of the pooled effect of the meta-analysis. ORs with 95% CI were calculated to assess outcome difference during and after HIHR or LIHR/OIHR. To evaluate heterogeneity, Q statistics and I^2^ were calculated. Two-sided p-value below 0.05 was considered statistically significant.

## Results

### Search and study characteristics

**Fig. 1:**
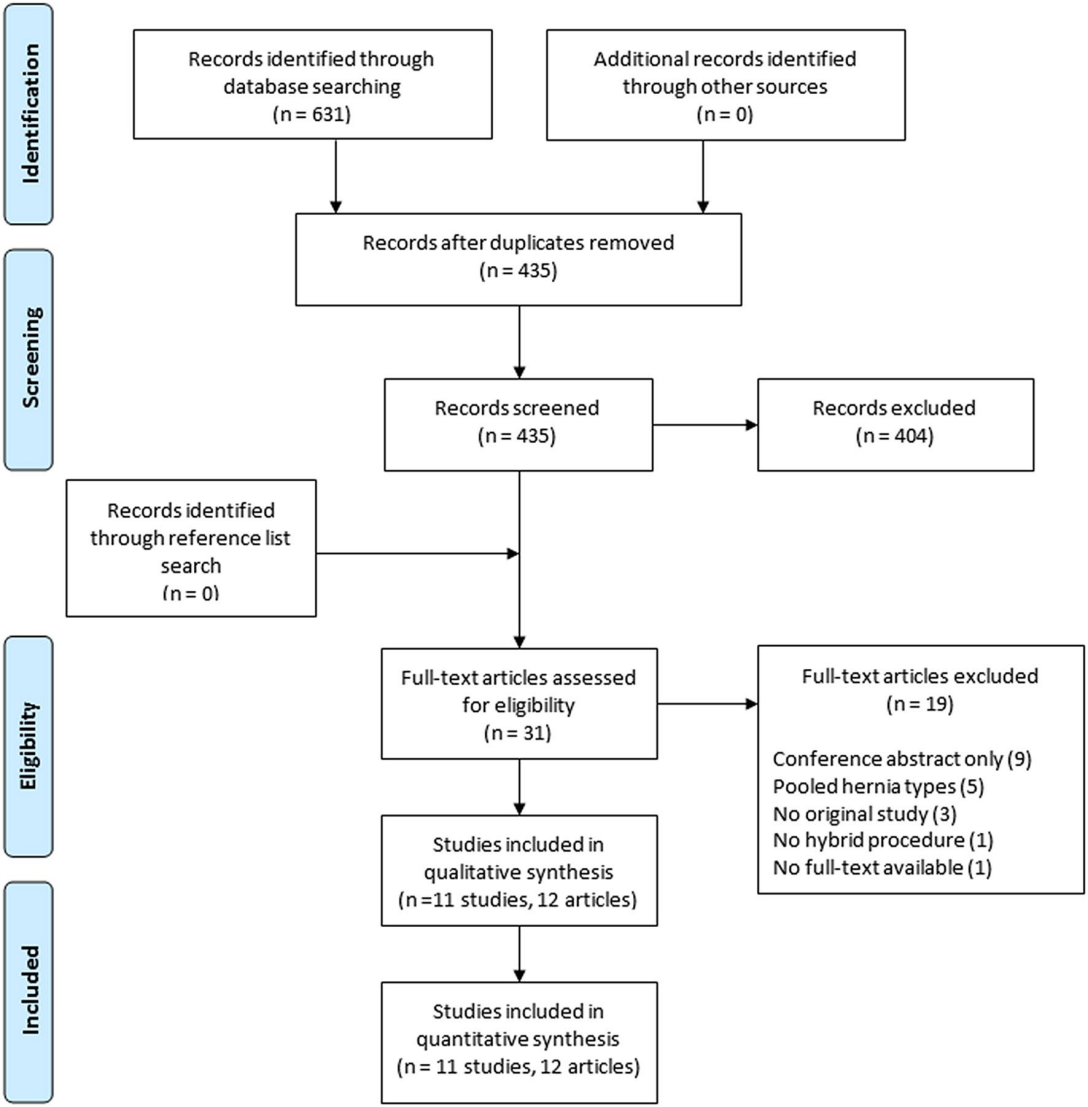
Preferred items for reporting of systematic reviews and meta-analyses (PRISMA) flow diagram

The full search results are shown in the PRISMA flow diagram (Fig. 1). After removal of duplicates, a total of 435 articles were identified. After screening on title and abstract, 30 articles were selected for full-text reading. After full-text reading, 12 articles were judged eligible and included for final data analysis, representing a total of 1681 patients. Two articles were the short- and long-term outcomes of one randomized controlled trial of Ahonen-Siirtola et al*.* [10, 19]*,* and ten were retrospective cohort studies [20–29].

An overview of study characteristics including methodological quality of each included study is shown in Table 1. Patient and hernia details are shown in Table 2. Surgical details of included studies are presented in Table 3.

**Table 1:** Overview of included studies, study details

Author, year of publication	Design	LOE	Risk of bias / ROBINS-I	Comparison HIHR vs LIHR/OIHR
Ahonen-Siirtola et al. [20], 2017	Retrospective	2b	Moderate risk of bias	Yes, 24 vs 38
Ahonen-Siirtola et al. [10, 19], 2018 and 2020	RCT	1b	Moderate risk of bias	Yes, 90 vs 94
Amaral et al. [21], 2019	Retrospective	3b	Moderate risk of bias	No
Ozturk et al. [27], 2015	Retrospective	3b	Moderate risk of bias	Yes, 16 vs 12
Ji et al. [22], 2013	Retrospective	2b	Moderate risk of bias	No, EH vs LH^a^
Wasim et al. [24], 2020	Retrospective	2b	Moderate risk of bias	No
Reinpold et al. [26], 2018	Prospective^b^	2b	Low risk of bias	Yes, 541 vs 541
Halka et al. [25], 2017	Retrospective	2b	Low risk of bias	Yes, 25 vs 57
Van den Dop et al. [23], 2020	Retrospective	2b	Moderate risk of bias	No
Addo et al. [29], 2020	Retrospective	2b	Moderate risk of bias	Yes, 10 vs 55^c^
Kudsi et al. [28], 2021	Retrospective	3b	Moderate risk of bias	No

^a^Early hybrid versus late hybrid

^b^Registry-based propensity score matched study 1:1

^c^Compared with OIHR

*EH* early hybrid, *LH* late hybrid, *LOE* level of evidence, *NOS* Newcastle-Ottowa scale, *RCT* randomized controlled trial, *ROBINS*-I risk of bias in non-randomised studies – of interventions

**Table 2:** Overview of included studies, patient and hernia details

Author	Patient details						Hernia details		
	Number	Male gender	Age (years)	BMI (kg/m^2^)	Length of stay (days)	Follow-up in months	Type	Location	Size (cm or cm2)
Ahonen-Siirtola et al. [20], 2017	62	35.5%	H: 58 ± 11.4 L: 61 ± 12.7	H: 31.8 ± 5.6 L: 31 ± 6.3	H: 5 L: 4	~	IH	Midline	H: 30.8 ± 23 L: 40.2 ± 52.7
Ahonen-Siirtola et al. [10, 19], 2018 and 2020	184	44%	H: 60 ± 12.8 L: 57 ± 11.4	H: 29.2 ± 4. L: 30.2 ± 4.4	H: 3.1 ± 3.2 L: 2.4 ± 1.9	1 and 12	IH	Midline	H: 10.5 ± 8.9 L: 13.2 ± 11.1
Amaral et al. [21], 2019	16	38%	59 ± 8	29.5 ± 5	3	37	IH^a^	Flank	6.4 ± 2.8
Ozturk et al. [27], 2015	28	14.3%	H: 59 L: 57	H: 30 L: 30	H: 2.8 ± 3 L: 2.4 ± 2	12	IH	Midline	H: 12.8 ± 5 L: 12.5 ± 4
Ji et al. [22], 2013	42	50%	EH: 59.6 ± 11.8 LH: 58.4 ± 11.8	EH: 32.4 ± 4.7 LH: 30.7 ± 4.2	EH: 4.7 ± 1.9 LH: 6.1 ± 2.3	EH: 14.9 ± 10.3 LH: 27.4 ± 11.5	IH	Midline/Flank	EH: 190 ± 84.5 LH: 178.1 ± 75.8
Wasim et al. [24], 2020	30^b^	16%	42 (31–60)	28 (20–35)	2.5	24	IH	Midline	6.2
Reinpold et al. [26], 2018	1082	H: 54.5% L: 54.3%	H: 60.2 ± 13.1 L: 60.3 ± 13.3	H: 29.7 ± 6.1 L: 29.6 ± 5.8	~	12	IH	Midline	H: 75.6 ± 100.6 L: 78.3 ± 97.8
Halka et al. [25], 2017	82	64%	H: 61.5 ± 11.52 R: 58.1 ± 13.9	H: 33.6 ± 7.1 R: 34.7 ± 6.7	H: 3.7 ± 2.3 R: 2.8 ± 1.8	1	IH	Midline	H: 21.5 ± 7.1 R: 16.3 ± 5.8
Van den Dop et al. [23], 2020	70	34.3%	59 ± 12.0	30 ± 6.1	3.3 ± 3.0	3.25 ± 6.25	IH	Midline/Flank	4.8 ± 2.4
Addo et al. [29], 2020	65	38.5%	H: 65.1 ± 12.0 O: 56.2 ± 10.8	H: 32.0 ± 7.6 O: 33.5 ± 6.8	H: 3.6 ± 1.3 O: 5.3 ± 2.3	H: 12.3 O: 12.6	IH	Midline	H: 14.4 ± 6.6 O: 13.6 ± 5.8
Kudsi et al. [28], 2021	20	55%	64 – 11.5	33.5 – 4.4	1.8	10.6	IH	Midline	15 (19.5 – 22.5)

Continuous data are median (interquartile range), mean (standard deviation) or mean (standard deviation, range)

^a^One of the 16 patients had a primary hernia

^b^Thirty of the 75 patients had an incisional hernia

~ Not specified

*BMI* body mass index, *EH* early hybrid, *H* hybrid, *IH* incisional hernia, *L* laparoscopic, *LH* late hybrid,* R *robotic

**Table 3:** Surgical details of included studies

Author	Mesh position	Mesh type	Mesh fixation	Operation time (min)
Ahonen-Siirtola et al. [20], 2017	IPOM	~	Sutures & Tackers	H: 134 ± 38 L: 128 ± 57
Ahonen-Siirtola et al. [10, 19], 2018 and 2020	IPOM	Parietex^®^ composite mesh, Covidien	Sutures & Tackers	H: 84 ± 29 L: 81 ± 46.7
Amaral et al. [21], 2019	IPOM and Onlay	Intraperitoneal coated synthetic mesh and uncoated polypropylene mesh	Tackers	159 ± 40
Ozturk et al. [27], 2015	IPOM	Polypropylene finned mesh	Sutures & Tackers	64.8 ± 23.2
Ji et al. [22], 2013	IPOM	EPTF mesh	Sutures & Tackers	H: 77.3 ± 35 L: 76.4 ± 32
Wasim et al. [24], 2020	IPOM	Lightweight composite mesh	Sutures & Tackers	60 (60–80)
Reinpold et al. [26], 2018	Sublay	Large pore standard alloplastic mesh, polypropylene or PVDF	Absorbable sutures if needed	103 (40–332)
Halka et al. [25], 2017	Sublay	Parietene, BARD, Versatex	Suture if needed	H: 345 R: 317
Van den Dop et al. [23], 2020	IPOM	Ventralight^®^, Prolene^®^ and Phasix^®^	Sutures & Tackers	100 ± 44.8
Addo et al. [29], 2020	Sublay	Polypropylene mesh	No fixation used	H: 294.5 ± 66.0 O: 267.5 ± 67.9
Kudsi et al. [28], 2021	Sublay	Synecor Pre™	No fixation used	296.5 ± 94.5

Continuous data are median (interquartile range), mean (standard deviation) or mean (standard deviation, range)

~ Not specified

*EH* early hybrid, *H* hybrid, *IPOM* intraperitoneal onlay mesh, *L* laparoscopic, *LH* late hybrid, *R* robotic

### HIHR techniques

All studies included performed a combination of open and laparoscopic approach. An extensive description of the surgical techniques is presented in Supplemental Table 1.

In brief, Ahonen-Siirtola and colleagues [10, 19, 20] (114 patients) and Ozturk et al*.* [27] (16 patients) described in their HIHR that they started with a (mini-)laparotomy to facilitate fascial defect closure before performing laparoscopic mesh fixation.

Amaral et al. [21] used a HIHR technique in sixteen patients that commenced with a laparoscopic adhesiolysis part, continued with a laparotomy for mesh placement, and laparoscopic fixation of the mesh in the final part. This sequence is also used by Van den Dop et al*.* [23] in 70 patients, Kudsi et al*.* [28] in 20 patients, by Ji et al*.* [22] in 42 patients and by Wasim et al*.* [24], in 30 patients.

The HIHR technique by Halka et al*.* [25] used in 25 patients and Kudsi and colleagues [28] in 20 patients was characterized by a combination of a robotic and open procedure for their transverse abdominis release technique. They start with robotic adhesiolysis, transverse abdominis release, and closing of the posterior fascia. They continue with a laparotomy and excise the hernia sac, excess skin and soft tissue before placing the mesh. Mesh placement is performed in the retro-rectus position, without the need for potentially traumatic mesh fixation. Sutures can be used if considered required. The skin and subcutaneous layers are then closed with absorbable sutures. The same surgical technicality is executed by Addo et al*. *[29], but they also perform intra-abdominal adhesiolysis prior closing of the posterior rectus fascia, in ten patients.

Reinpold and colleagues [26] utilized a signature technique in 541 patients which they named the MILOS (Mini- or Less Open Sublay Operation). The authors started with a laparotomy above the centre of the hernia defect, and placed a laparoscope inside the hernia defect, after which adhesiolysis was performed. Circumferentially, the posterior rectus sheath was mobilized from the rectus muscle for mesh placement in the sublay position. Alternatively, the mesh could also be placed in the preperitoneal plane. Analogous to the technicalities of Addo, Kudsi and Halka, mesh fixation can be performed atraumatic. The main hernia defect was closed with minimal tension above the mesh, and large hernia sacs were excised. The skin was closed with running subcutaneous sutures.

### Intra-operative complications

Eight studies, composed of 1506 patients, reported intra-operative complications with an incidence ranging from 0 to 16.7%, including 28 enterotomies, 20 bleedings and one bladder injury (Table 4). Three studies [19, 20, 26], representing 1328 patients, compared HIHR with LIHR. There was no statistically significant difference in intra-operative complications between HIHR (14 of 651 (2.2%)) and LIHR (22 of 677 (3.3%); odds ratio = 0.63 (95% CI 0.34–1.18); I^2^ = 51%; *p* = 0.15) (Supplemental Fig. 1).

**Table 4:** Intra-operative complications

Author	Enterotomy	Bladder injury	Bleeding	Total intra-operative complications
Ahonen-Siirtola et al. [20], 2017	H: 4 (16.7%) L: 5 (13.2%)	0	0	H: 4 (16.7%) L: 5 (13.2%)
Ahonen-Siirtola et al. [10, 19], 2018 and 2020	H: 1 (1.1%) L: 5 (5.3%)	H: 1 (1.1%) L: 0	H: 4 (4.4%) L: 1 (1.1%)	H: 6 (6.6%) L: 6 (6.4%)
Amaral et al. [21], 2019	0	~	~	0
Ozturk et al. [27], 2015	~	~	~	~
Ji et al. [22], 2013	EH: 0 LH: 6 (29%)	0	EH: 0 LH: 2 (9.5%)	EH: 0 LH: 8 (38.1%)
Wasim et al. [24], 2020	0	0	0	0
Reinpold et al. [26], 2018	H: 1 (0.2%) L: 3 (0.6%)	0	H: 3 (0.6%) L: 9 (1.7%)	H: 4 (0.7%) L: 12 (2.2%)
Halka et al. [25], 2017	~	~	~	~
Van den Dop et al. [23], 2020	2 (2.8%)	0	1 (2.8%)	5 (7.0%)
Addo et al. [29], 2020	~	~	~	~
Kudsi et al. [28], 2021	1 (5%)	0	0	1 (5%)

~ Not specified

*EH* early hybrid, *H* hybrid, *L* laparoscopic, *LH* late hybrid

### Postoperative complications

**Fig. 2:**
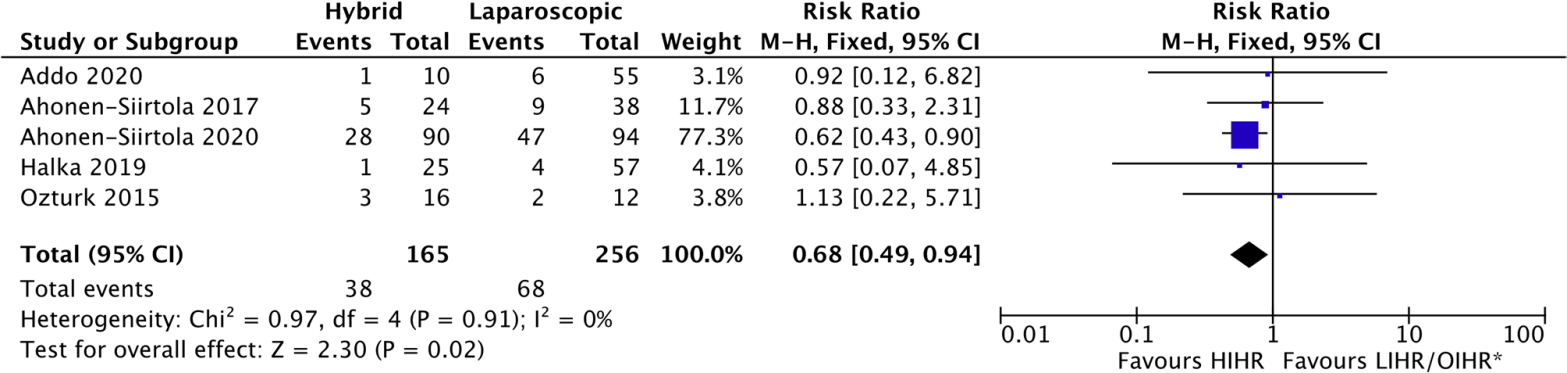
Forest plot SSOs occurring after HIHR versus LIHR. M-H, random = Mantel–Haenszel random-effects model; df = degrees of freedom. *Addo 2020 compared HIHR and OIHR

One-hundred-and-sixty of all 1681 (9.5%) patients had postoperative complications (Table 5). Five studies [19, 20, 25, 27, 29], representing 421 patients, compared SSOs between HIHR and LIHR or OIHR and were included in the meta-analysis (Fig. 2). In the case of Addo and colleagues [29], HIHR was compared to OHIR. The SSO rate was significantly lower in the HIHR group (38 of 165 (23.0%)) compared to the LIHR or OIHR group (68 of 256 (26.6%); odds ratio = 0.68 (95% CI 0.49–0.94); I^2^ = 0%; *p* = 0.02). When removing the study of Ahonen-Siirtola et al. [10], the difference in SSO between HIHR and LIHR was no longer statistically significant (HIHR group (10 of 75 (13.3%)) compared to the LIHR or OIHR group (21 of 162 (13.0%); odds ratio = 0.87 (95% CI 0.42–1.79); I^2^ = 0%; *p* = 0.71)). Seroma formation was reported in five studies comparing HIHR and LIHR or OIHR [19, 20, 26, 27, 29], where it was found to occur significantly less frequent in the HIHR group (36 of 681 (5.3%) versus 77 of 740 (10.4%); odds ratio = 0.40 (95 CI 0.25–0.64; I^2^ = 40%, *p* < 0.001) (Supplemental Fig. 2). Excluding every study one by one did not alter the p-value above 0.05.

**Table 5:** Postoperative complications

Author	Seroma	SSO	SSOPI	Pain (VAS)	Hematoma	Readmission	Reoperation	Mortality	Total complications^c^
Ahonen-Siirtola et al. [20], 2017	H: 4 (16.7%) L: 6 (15.8%)	H: 5 (20.9%) L: 9 (23.7%)	0	~	~	~	H: 0 L: 4 (10.5%)	H: 0 L: 1 (2.6%)	[H: 6 (25%) L: 11 (28.9%)]
Ahonen-Siirtola et al. [10, 19], 2018 and 2020	**1 m**: ^a^H: 27 (31.4%) L: 46 (48.9%) **1y**: ^b^H: 5 (6%) L: 12 (13%)	**1 m**: ^a^H: 28 (32.5%) L: 47 (50%) **1y**: ^b^H: 5 (6%) L: 12 (13%)	H: 0 L: 6 (6.4%)	**1 m**: H: 2.22 ± 1.51 L: 2.19 ± 1.84 **1y**: H: 1.4 ± 1.1 L: 1.5 ± 1.2	H: 4 (4.4%) L: 4 (4.3%)	H: 3 (3.3%) L: 13 (13.8%)	H: 4 (4.4%) L: 1 (1.1%) Rec: H: 1 (1%) L: 1 (1%)	H: 1 (1.1%) L: 0	[H: 11 (12.2%) L: 15 (16%)]
Amaral et al. [21], 2019	2 (12.5%)	2 (12.5%)	0	~	0	~	~	~	{2 (12.5%)}
Ozturk et al. [27], 2015	H: 1 (6.3%) L: 4 (33.3%)	H: 3 (18.8%) L: 2 (16.6%)	H: 0 L: 1 (8.3%)	~	0	~	~	~	[H: 6 (37.5%) L: 6 (50%)]
Ji et al. [22], 2013	0	~	0	~	0	EH: 0% LH: 1 (4.8%)	EH: 0% LH: 1 (4.8%)	~	EH: 4 (19%) LH: 11 (52%)
Wasim et al. [24], 2020	1 (3%)	1 (3%)	0	2	0	0	0	0	{1 (3%)}
Reinpold et al. [26], 2018	H: 3 (0.6%) L: 18 (3.3%)	~	H: 9 (1.7%) L: 18 (3.3%)	~	~	~	H: 9 (1.7%) L: 18 (3.3%)	~	{H: 7 (1.3%) L: 13 5.7%}
Halka et al. [25], 2017	~	H: 1 (4%) R: 4 (7%)	H: 1 (4%) R: 4 (7%)	~	H: 0 R: 2 (3.5%)	~	H: 0 R: 1 (1.8%)	~	[H: 10 (40%), R: 16 (28%)]
Van den Dop et al. [23], 2020	2 (2.8%)	1 (1.4%)	0	~	1 (1.4%)	~	0	0	[18 (25.7%)]
Addo et al. [29], 2020	H: 1 (10%) O: 3 (5.5%)	H: 1 (10%) O: 6 (10.9%)	~	~	H: 0 O: 3 (5.5%)	H: 0 O: 4 (7.5%)	H: 0 O: 5 (9.1%)	~	[H: 1 (10%) O: 15 (27.3%)]
Kudsi et al. [28], 2021	3 (15%)	5 (25%)	2 (10%)	~	~	2 (10%)	0	0	[7 (35%)]

^a^Clinical findings

^b^Radiological findings

^c^[Total percentage of complications per cohort (patients could present themselves with multiple complications)] or {total percentage of patients with complications per cohort}

~ Not specified

*1 m* 1 month, *1y* 1 year, *EH* Early Hybrid, *H* Hybrid, *L* Laparoscopic, *LH* Late Hybrid, *Rec* recurrence

**Fig. 3:**
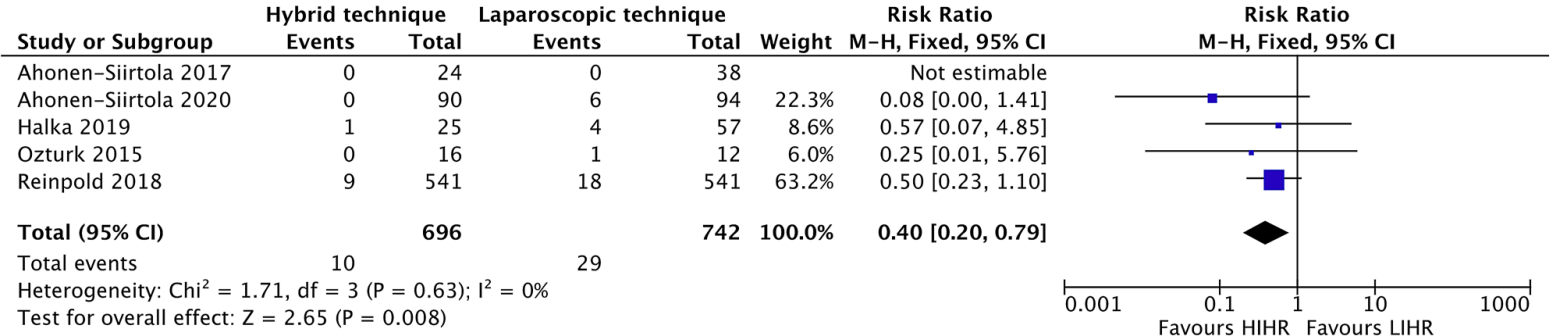
Forest plot of SSOPIs occuring after HIHR versus LIHR. M-H, random = Mantel–Haenszel random-effects model; df = degrees of freedom

Five studies [19, 20, 25–27], representing 1376 patients, compared SSOPIs between HIHR and LIHR and were included in the meta-analysis (Fig. 3). The SSOPI rate was significantly lower in the HIHR group (10 of 672 (1.5%)) compared to the LIHR group (29 of 704 (4.1%); odds ratio = 0.39 (95% CI 0.19–0.78); I^2^ = 0%; *p* = 0.008) (Fig. 3). Excluding every study one by one did not augmented the p-value above 0.05.

## Discussion

This systematic review found a relatively low incidence of intra- and postoperative complications after HIHR. Compared to LIHR or OIHR in a meta-analysis, statistically significant lower odds of developing a SSO, formation of seroma and SSOPIs were found in the HIHR group. Intra-operative complications also seem to occur less frequent during the HIHR compared to the LIHR, although no statistically significant difference was found.

In IH repair, postoperative complication rates can reach up to 60% [9]. However, this current systematic review showed a relative low rate of overall postoperative complications after HIHR. A possible explanation for the lower rate of SSOs, seroma and SSOPIs after HIHR could be due to the fact that this technique offers a broader overview of the hernia defect when using both laparoscopic and open approaches. For instance, the seroma formation observed in LIHR is reported to be 13% after 1 year and 6% in HIHR in the study by Ahonen et al*.* [10]. Other studies report seroma formation rates of 7–28% [30–32]. Seroma formation found with the hybrid procedures ranges from 0 to 16%, which could be explained by the effect of complete resection of the hernia sac being easier when using HIHR. The effect of resection of the hernia sac on seroma formation has been investigated before, hernia sac resection being recommended by the EuraHS working group [33]. Another important postoperative complication after IH repair is acute and chronic pain. While laparoscopic IPOM fixation is associated with a higher incidence of acute and chronic pain compared to sublay mesh fixation [34], the heterogeneity in pain reporting in the included studies prevented current study from making unequivocal comparisons between these two mesh fixations.

Bowel injury is one of the most feared intra-operative complication during LIHR. The incidence of enterotomies during LIHR is ranging from 0 to 14% in a review of LeBlanc et al*.* [35]. When any enterotomy is detected during the procedure, the bowel defect will be sutured, and depending on the surgeon`s preference, an absorbable biological mesh will be placed or the defect will be closed primary with sutures. Placing a non-absorbable synthetic mesh into a contaminated field increases the risk of mesh infection, while primary closing with sutures is known to increase the chance of recurrence. All the more, while these recognized enterotomies place the surgeon in a predicament, there is also a chance that a bowel injury remains undetected until the patient presents signs of peritonitis postoperatively. Ahonen-Siirtola et al*.* [20] described four cases where bowel injury remained undetected in the LIHR group, after which one patient died in the following weeks from the consequences of septic shock. No undetected bowel injury occurred in the HIHR group. Early recognition of these enterotomies seems, therefore, of paramount importance.

HIHR should be considered in the light of an additional surgical modality rather than a different operative technique. To optimize the strategy for IH repair, the two approaches can be combined, and more options become available to tackle the IH. This optimization of strategies could be achieved by amplifying the advantages of both the open approach (e.g. complete resection of hernia sac, safe adhesiolysis, optimal closure of fascia, skin reduction and scar correction, commodious bowel examination for perforations) and laparoscopic approach (optimal view to detect adhesions and hernia configuration, intra-abdominal placement and fixation of mesh) while disadvantages of both procedures (e.g. larger incision than necessary with open approach, seroma formation with laparoscopic approach) could be curtailed. An underexposed advantage of HIHR is the cosmetic outcome. Though not investigated as an outcome in this review, compared to LIHR, HIHR gives an option to perform any reduction of redundant skin and scar correction, with possibly higher satisfactory cosmetic results. Mesh placement during HIHR techniques was predominantly in the IPOM or sublay position. Although no reliable comparisons between mesh positions could be drawn from the articles in this study, it is appreciable that optimal mesh positioning during HIHR may warrant further exploration.

In recent years, surgeons have been searching for the best surgical procedure for IH repair with the development of various new surgical techniques as a result [36]. One explanation for this growing interest is the senescence of the general patient population with concomitantly increased comorbidities. Especially patients with IH are known to have more comorbidities like older age, larger defects and diabetes compared to the patients with a primary ventral hernia, increasing the need for a recurrence avoidable technique for patients with these hernias [37, 38]. HIHR offers the benefits of both open and laparoscopic approach, making this technique a more suitable choice to use for difficult IH and patients with comorbidities. In fact, it is quite possible that the single-arm cohort studies included in this review have deliberately selected a frail patient population to undergo HIHR.

## Limitations

This systematic review has several limitations. The high level of heterogeneity in surgical procedures and reporting on postoperative complications are important limitations of this review. The I-squared test can result in biased results when a small number of studies are included or when the confidence intervals of included studies are large, as is the case in current meta-analysis with an I^2^ of 0. The definition of a hybrid procedure in this study was made to form a more homogenous group, though much variation still exists after including studies for using a combination of the open and laparoscopic approach. The incidence of SSOs was found to be not statistically significant after excluding the article of Ahonen-Siirtola et al., indicating a strong pooled incidence. Furthermore, the level of evidence of the included studies was relatively low and most studies were retrospective and consisted of a single-arm cohort, withholding to include these studies and compare surgical techniques in a meta-analysis. Accordingly, the outcomes have to be interpreted with caution.

## Conclusion

HIHR is promising with respect to intra- and postoperative complications, leading seemingly to less SSOs, seroma and SSOPIs. Prospective RCTs are needed to make statements of the feasibility and effectiveness of HIHR. This systematic review forms a strong invitation for RCTs to confirm the benefits of this approach.

## Supplementary Information

Below is the link to the electronic supplementary material.

Supplementary file1 (PNG 102 KB)

Supplementary file2 (PNG 374 KB)

Supplementary file3 (DOCX 28 KB)

## Data Availability

All data and material are stored on a computer in the local hospital.

## Code availability

Review Manger 5.3 has been used to do all calculations in this article.
