# Supplementary figures and images for: Hybrid operation technique for incisional hernia repair: a systematic review and meta-analysis of intra- and postoperative complications
Source: Hernia. 2021 Sep 18;25(6):1459–69. doi: 10.1007/s10029-021-02497-3 (PMC8613158; doi:10.1007/s10029-021-02497-3)

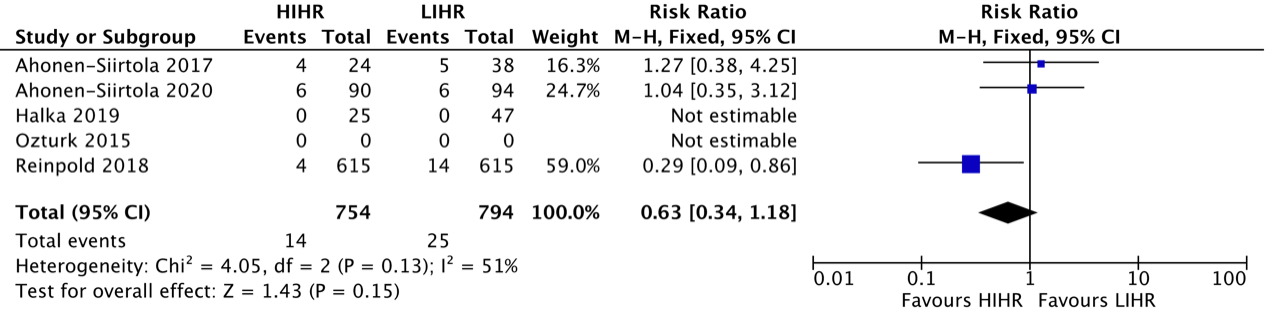

Supplement: Supplementary file 1 — Supplementary file1 (PNG 102 KB) [file 10029_2021_2497_MOESM1_ESM.png]

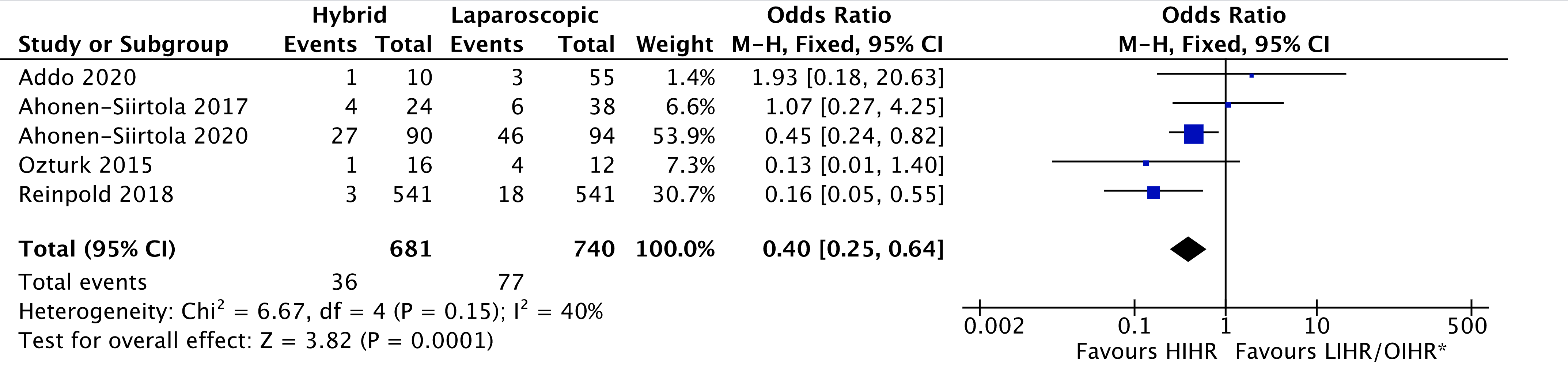

Supplement: Supplementary file 2 — Supplementary file2 (PNG 374 KB) [file 10029_2021_2497_MOESM2_ESM.png]
