# Supplementary material for: Hybrid operation technique for incisional hernia repair: a systematic review and meta-analysis of intra- and postoperative complications
Source: Hernia. 2021 Sep 18;25(6):1459–69. doi: 10.1007/s10029-021-02497-3 (PMC8613158; doi:10.1007/s10029-021-02497-3)
Supplement: Supplementary file 3 — Supplementary file3 (DOCX 28 KB) [file 10029_2021_2497_MOESM3_ESM.docx]

**Supplemental Table 1.** Description of surgical procedure of each included study.

| Author, year of publication | Description of surgical procedure |
| --- | --- |
| Ahonen-Siirtola, 2017 | Hybrid approach included a mini-laparotomy phase during which adhesiolysis was performed and the fascia defect was closed before laparoscopic mesh fixation. |
| Ahonen-Siirtola, 2018 and 2020 | The hernia was resected, and the fascial defect was closed with a slowly absorbing monofilament suture through a mini-laparotomy incision before the standard mesh repair. |
| Amaral 2019 | First laparoscopic part where routinely all adhesions had been taken down between intra-abdominal contents (…) the second open part where a transverse incision of 8-10 cm is made over the defect where after a mesh is played in onlay position (…) the final laparoscopic part where a mesh is fixated in IPOM manner with tackers and sutures |
| Ozturk 2015 | Fascial defect was exposed via incision over previous surgical scar and hernia sac incised to access into the abdomen. Adhesions were dissected and three laparoscopic trocars were inserted into the abdomen as mentioned above under direct vision without using Versaport. EPTF mesh was laid under the fascial defect in abdomen and six prolene fixation sutures were used at each side. Then, mesh material left in abdomen and fascial defect was primarily closed. Afterwards, laparoscope inserted and mesh material was fixed to inner abdominal wall via tacker. |
| Ji 2013 | After introduction of the laparoscope, the adhesions of the bowel to the abdominal wall were evaluated (…) under laparoscopic guidance, a 5- to 10-cm incision was made over the hernia defect adjacent to the area with dense adhesions. After adhesiolysis, four to eight sutures were placed in the perimeter of the mesh, and both tails of the suture were left long. Pneumoperitoneum was reestablished and the mesh was fixated using tackers and the sutures to the abdominal wall. |
| Wasim2020 | The hybrid techniques used were (1) Laparoscopic adhesiolysis, open sac excision with closure of defect and a laparoscopic mesh placement. This was done in 50 patients, with large hernial sac. Harmonic shears or bipolar was used for laparoscopic adhesiolysis. Contents of hernia sac were reduced. A small circumumbilical incision was taken, and complete hernia sac was excised. The hernia defect was closed with interrupted prolene sutures and skin closed with moncryl.  (2) Laparoscopic adhesiolysis, omphalectomy with closure of defect and laparoscopic mesh placement. This was done in 15 patients, with large sac, multiple defects and necrotic umbilical skin. Laparoscopic adhesiolysis was done, and hernia contents were reduced. The abdomen was desufflated and omphalectomy was done. Hernia sac was excised, and the defect was closed with interrupted prolene sutures and skin closed with moncryl.  (3) Open adhesiolysis, sac excision with closure of defect and laparoscopic mesh placement. This was done in 10 patients with large hernia sac with small defect and obstructed bowel. Diagnostic laparoscopy was performed. The abdomen was desufflated and infraumbilical incision was taken; open adhesiolysis was done. Hernia sac was excised, and the defect was closed with interrupted prolene sutures and skin closed with monocryl. |
| Reinpold, 2018 | Step 1: The MILOS operation starts with a 2 to 12 cm skin incision (2–5 cm ¼ mini-open, 6–12 cm ¼ less open) directly above  the center of the hernia defect, followed by complete exposure of the hernia sac. Step 2: A small incision of the hernia sac for transhernial laparoscopy is advisable. If necessary, this is followed by laparoscopic or mini-open adhesiolysis. Excessive parts of the hernia sac which pose a risk of bowel obstruction are excised. Step 3: The border of the hernia defect (hernia ring) is circumferentially exposed and elevated with sharp clamps. Step 4: The peritoneum is detached from the abdominal wall at the edge of the fascia defect with a radius of at least 2 cm. Step 5: The posterior rectus sheath is incised on both sides about 1 cm lateral to the medial border of the rectus muscle. Step 6: The abdominal wall is elevated by the assistant using pairs of narrow retractors of different size around the hernia defect. In the midline, the peritoneum is separated from the linea alba. The posterior rectus sheath is extensively mobilized from  the rectus muscle with laparoscopic instruments. Dissection is performed circumferentially around the hernia defect either under direct visualization or endoscopic view using laparoscopic instruments armed with a 10-mm light tube which was  specifically designed by our working group and Wolf company. The Endotorch is a modified 20 cm long and 10 mm diameter laparoscope. Instead of a telescopic rod lens system it has a central canal for the insertion of any 5 mm laparoscopic instrument. The detailed features of the Endotorch will be described in a separate publication. Before the introduction of the Endotorch in 2014, an endoscopic light cable was attached parallel to a laparoscopic instrument. The Endotorch gives maximum light at the tip of the light  holding laparoscopic instrument, thus automatically pointing to the center of the surgeon’s dissection field. This allows precise  wide range tissue manipulation via mini incisions within the extra peritoneal space. It may also be used for gasless laparoscopy and  adhesiolysis. The circumferential dissection range in relation to the skin incision and recommended size of the rectangular retractors are  given in Table Supplement 3. MILOS operations via 2 cm incisions were performed with 3 mm laparoscopic instruments and a 5 mm laparoscope. Scar tissue formation, especially after previous operation(s) with mesh implantation, may reduce the maximum dissection range and warrant larger incisions. Step 7: The posterior layer of the rectus sheath is longitudinally incised in all quadrants, about 1 cm lateral to the medial border of the rectus muscle corresponding to the size of the hernia defect and planned alloplastic mesh insertion. After creation of an extraperitoneal space of at least 8 cm in diameter and closure of the peritoneum, the operation may be converted to total extraperitoneal gas endoscopy (endoscopic TEP, ventral hernia repair). Reusable standard ports, or a transhernial single port technique may be used. After temporary hernia defect closure with a running suture one 10 mm optic trocar and one 5 mm working port are inserted via the end points of the suture line without additional skin incisions. After extraperitoneal CO2 application with a maximum pressure of 12 mmHg, a second 5 mm working trocar is inserted through the rectus muscle at least 5 cm lateral to the optic port for better angulation. The posterior layer of the rectus sheath is closed if this is possible with low tension. In all other cases, defects of the peritoneum between the cut edges of the posterior rectus sheath are meticulously closed to prevent any contact between alloplastic material and the intestines. Step 8: A large pore standard alloplastic mesh, preferably polypropylene or polyvinylidenfluoride (PVDF) is double rolled and inserted transhernially with 2 long curved clamps without skin contact and then unfolded with light-armed laparoscopic instruments under direct or endoscopic vision. The mesh should posteriorly overlap the hernia defect by at least 5 cm. The implantation of verylarge meshes is possible. In most cases, because of large overlap, there is no need for mesh fixation. In the case of subxiphoidal or suprapubic hernia defects, the mesh is secured with absorbable sutures to the paraxiphoidal fascia or Cooper’s ligaments. Fascial circumferential lateral mesh fixation with absorbable sutures is only performed if a low tension hernia defect closure is not possible (bridging of the hernia defect). One suction Redon drain (8 Charr.) is inserted into the extraperitoneal space. Step 9: Additional hernia defects are closed transhernially under direct vision or endoscopically. The main hernia defect is closed with minimal tension above the mesh. Anatomical reconstruction of the abdominal wall is always the primary goal. Step 10: Management of subcutaneous tissue and skin: Large hernia sacs are removed, meticulous subcutaneous electrocoagulation is performed and a subcutaneous 8 Char. Redon drain is inserted. If necessary, contracted scar tissue is mobilized and resected, and the umbilicus is reconstructed. The skin is closed with a running subcutaneous suture. |
| Halka 2017 | The abdomen is entered laparoscopically and robotic ports are placed along the anterior axillary line. After reducing hernia contents and performing necessary adhesiolysis, a retrorectus dissection is performed to the semilunar line whereupon the posterior lamella of the internal oblique is incised. The transversus abdominis muscle is then divided and a plane is developed between the transversus abdominis muscle and the transversalis fascia. This dissection is performed laterally to the posterior axillary line, superiorly to the central tendon of the diaphragm, and inferiorly to the space of Retzius as needed. The robot is then re-docked on the contralateral side and an identical dissection is performed. The posterior fascia is then closed with barbed, absorbable suture. Following this dissection, an incision is made through the hernia sac; the entire hernia sac, excess skin and soft tissue, and any prior mesh is then excised. The majority of incisions were vertical unless the patient had a previous off-midline incision. The new mesh is then placed with care taken to ensure it lies flat and is of adequate size relative to the hernia defect. In defects extending below the umbilicus the mesh was affixed to Cooper’s ligament with prolene, otherwise we used no transfascial sutures. The anterior fascia is then re-approximated with a continuous running heavy PDS suture. Three brands of mesh were used in both the hybrid and open operations: parietene (Medtronic), Bard Mesh (Bard), and Versatex (Medtronic). The choice of mesh was left to the surgeon’s discretion based on hernia characteristics. The skin and subcutaneous tissue are then closed in layers with absorbable suture. Retromuscular drains are placed in all patients. |
| Van den Dop, 2020 | First, a “pneumodissection” of the hernia sac was formed with the introduction of a Veress needle in the intraabdominal space on the left subcostal regionThis pneumodissection was formed when pressure of the CO2 intraperitoneally expands the abdomen and the hernia sac would bulge through the abdominal wall and thereby aiding in determining the length of the laparotomy incision. Concomitantly, this would facilitate a safer way for adhesiolysis, gave way for more preservation of abdominal wall, smaller laparotomy incision and optimised the surgical plan for mesh implementation. A 12 mm visual port was inserted under vision on the contralateral side from the location of the abdominal wall hernia, in case of a midline hernia on de left lateral side, together with two 5 mm trocar ports, both on the contralateral side. The laparoscope was then introduced to inspect the abdominal cavity and identify the hernia sac. Laparoscopic start of adhesiolysis was performed for adhesions around the abdominal defect for safe continuation of the mini-laparotomy. Secondly, the laparoscopic procedure was interrupted, the abdomen deflated and a minimal length excision of the old scar was performed. The dissection was continued through the subcutaneous fat and access to the extraperitoneal space was obtained. The hernia sac was opened, open adhesiolysis was performed if laparoscopic adhesiolysis was deemed too dangerous because of unclear view and potential contents of the hernia sac were repositioned in the intra-abdominal space. After excision of the hernia sac, the edges of the fascia were exposed circumferentially. In case of an Echo Positioning System was used, the mesh was put in through the incision and the posterior rectus fascia was closed with PDS (polydiaxone) loop sutures. Thirdly, the procedure was continued laparoscopically, the abdomen inflated and in the case of a Ventralight ST mesh, the mesh was now introduced into the abdominal cavity through the laparoscopic port. With the use of a Sorbafix tacker, the mesh was fixated to the abdominal wall. When using the Echo mesh, a disposable titanium body was fixated on the mesh. Through the expansion of the titanium body inside the abdomen, the mesh could easily be positioned in the centre of the closed hernia defect and ensure that the mesh surface was spread evenly. After the fixation in a single or double crown manner, the titanium body was evacuated. In case of use of a Ventralight ST mesh, 4 transfascial sutures with resorbable polyglactin were used for correct positioning before tackering. Lastly, a laparoscopic overview was used to ensure correct mesh position. The trocars and Veress needle were always removed under vision, closed suction drains were placed under the subcutaneous space if the woundsurface deemed considerably of size. The subcutaneous fat was approximated with Vicryl and the skin was sutured with resorbable monofilament sutures. |
| Addo 2020 | Depending on the size of the defect and planned reconstruction, we either enter the abdomen using an eTEP technique (for planned retrorectus dissection or retrorectus dissection with planned unilateral TAR) or a transabdominal technique for planned bilateral transversus abdominis releases. Either the laparoscopic or robotic approaches are used for this technique. For extraperitoneal approaches, the ipsilateral retrorectus space is developed and then a crossover is performed which enters the preperitoneal space and then subsequently the contralateral retrorectus space. The contralateral space is developed and if a TAR is needed on that side then a “top-down” or “bottoms-up” TAR is performed. When deemed appropriate, the intra-abdominal space is entered by incising the posterior layer, adhesiolysis is performed as needed. Posterior layer dissection is identical to open approach. Any defects in the posterior layer are closed with a 2–0 barbed suture. An incision is then made over the defect. During this step atrophic skin and scar are excised as needed but subcutaneous space is not developed to preserve subcutaneous perforators. Fascial edges of the defect are exposed. Mesh is placed generously overlapping the visceral sac. A retromuscular drain is placed through one of the port sites. The anterior fascial edges are re-approximated using 0 PDS monoflament suture. Subcutaneous drain is placed when a large subcutaneous space is appreciated. Retromuscular drain is removed 3–7 days after surgery. If subcutaneous drains are placed, then these are taken out at the 1 week follow-up if the output meets the criteria as discussed previously or left for an additional week |
| Kudsi 2021 | Preoperative consideration. For large incisional ventral hernias there is a high degree of complexity and judicious physical examination and preoperative imaging is necessary. Ideally, risks factors that affect wound healing such as obesity, malnutrition, and diabetes mellitus should be optimized, and smoking cessation should be encouraged before elective procedures. Obtaining recent abdominopelvic imaging with computed tomography allows for cross-sectional and longitudinal images that are valuable to delineate the hernia. Patient preparation. The patient is placed in the supine position (Fig. 1). Flexing the bed slightly allows for maximal space between the costal margin and iliac crest so as to lessen robotic arm interference. rRM VHRs were conducted through either a TA or totally extraperitoneal (TEP) access. Port access and positioning TA access. Initial access is obtained by inserting a Veress needle in the left upper quadrant (LUQ) (Fig. 2). An 8 mm port is inserted as lateral as possible within the rectus sheath in the LUQ. Two additional 8-mm ports are next inserted bluntly to prevent inadvertent bleeding, with an 8-cm spacing between ports. Adhesions are lysed as required. The posterior rectus fascia is then cut. The contralateral retrorectus dissection is first performed. Once this is completed, a TAR is performed as described earlier. The robot is undocked and three trocars are inserted similarly on the contralateral side. TEP access. The first trocar is inserted through optical trocar entry in the LUQ, as lateral as possible within the rectus sheath. A 5-mm laparoscopic 0 camera is inserted into a 5-mm optical trocar. Under direct visualization, the trocar is advanced through the subcutaneous tissue and the anterior rectus sheath. Immediately after visualizingthe rectus muscle fibers,the trocar is directed inferiorly to avoid penetration of the posterior layer. After usinglaparoscopic blunt dissection aided by high-pressure insufflation to dissect a sufficient area, the two remaining 8-mm trocars are inserted. With an 8-cm spacing between trocars, a lateromedial and angled advancement is used to insert trocars and minimize robotic arm mechanical interference. The initial 5-mm trocar is then substituted by an 8-mm trocar. Docking and instruments. Once the trocars are placed, the patient-side cart of the da Vinci surgical system (model Xi) is docked (Fig. 3). The following instruments are used: a bipolar grasper and monopolar scissors, which are later substituted by a needle driver. The 30 degree scope is directed toward the surgical field of interest and the system uses a predetermined algorithm to optimize arm positioning. RM dissection and crossover. Ipsilateral retrorectus dissection is performed and any previous transfascial sutures are cut if encountered (Fig. 4). Then a crossover technique is performed to reach its contralateral counterpart.11 In large incisional hernias, the tissue adjacent to the hernia are often fused and hard to differentiate. We recommend beginning crossover in virgin tissue planes away from the hernia, if possible. The medial border of the retrorectus space is incised to reach the contralateral side, followed by craniocaudal dissection toward the incisional hernia site. If a very thin preperitoneal layer is encountered, any tears in the posterior layer will need to be sutured later. After performing the crossover, both retrorectus spaces are now partially merged. Circumferential dissection is performed to facilitate hernia content reduction. The hernia sac is then breached to directly visualize reduction of the contents. To facilitate midline reconstruction and reinforce the visceral sac, a posterior component separation, TAR is performed. Transversus abdominis release. The transversus abdominis fibers are visualized along the contralateral rectus sheath (Fig. 5). Dissection is initiated along the contralateral rectus sheath, parallel and medial to the linea semilunaris (LS). It is crucial to avoid disruption of the LS, which is considered a technical error. Neurovascular structures are marked and preserved within the lateral edge of the retrorectus space. Next, the transversus abdominis muscle is separated from its fascia along the mid-axillary line using traction and conservative monopolar use. Under direct visualization, trocars are inserted in the contralateral right rectus space and TAR is performed similarly on the left side if needed. Fascial reconstruction. Reapproximation of the posterior rectus sheath edges is achieved with a 2-0 absorbable suture (Fig. 6). The anterior fascial defect closure is then started in a caudal to cranial direction, using absorbable barbed suture, taking 5–8 mm bites of the fascia with 5-mm spacing12 and leaving a gap to allow for mesh insertion. Loose suturing is used to begin hernia defect closure, which will later be tightened after mesh deployment. Mesh deployment. The skin is incised above hernia defect (Fig. 7). The mesh is inserted through the gap left during defect closure and is deployed to fully occupy the RM space. The remaining defect gap is closed through the open approach and the surgical field is re-insufflated, under low pressure, to assess the mesh positioning. Technical considerations. Mesh fixation is usually not required since the mesh fully occupies the RM space and intra-abdominal pressure helps maintain the mesh position. In general, drain placement is not required and fascial defects at the trocar sites are not closed. Skin incisions are sutured after injecting local anesthesia. In cases with redundant skin and soft tissue or hernia sac, these may be excised during the open portion of the hybrid technique. This technique can also address cases with open wounds due to mesh erosions |
